# Supplementary material for: Gastroprotection against Rat Ulcers by Nephthea Sterol Derivative
Source: Biomolecules. 2021 Aug 21;11(8):1247. doi: 10.3390/biom11081247 (PMC8393318; doi:10.3390/biom11081247)

Supplementary Materials

Gastroprotection against rat ulcers by *Nephthea* sterol derivitive

Tarik A. Mohamed [1], Abdelsamed I. Elshamy [2], Mahmoud A. A. Ibrahim [3], Mohamed A. M. Atia [4], Rania F. Ahmed [2], Sherin K. Ali [1], Karam A. Mahdy [5], Shifaa O. Alshammari [6], Ahmed M. Al-Abd [7,8,*], Mahmoud F. Moustafa[9,10], Abdel Razik H. Farrag [11], Mohamed-Elamir F. Hegazy [1]

[1] Chemistry of Medicinal Plants Department, National Research Centre, 33 El−Bohouth St., Dokki, Giza, 12622, Egypt; ta.mourad@nrc.sci.eg; me.fathy@nrc.sci.eg (M.E.F.H.)
[2] Chemistry of Natural Compounds Department, National Research Centre, Dokki, 12622 Giza, Egypt; ai.el-shamy@nrc.sci.eg (A.I.E.);
[3] Computational Chemistry Laboratory, Chemistry Department, Faculty of Science, Minia University, Minia 61519, Egypt; m.ibrahim@compche.net (M.A.A.I.)
[4] Molecular Genetics and Genome Mapping Laboratory, Genome Mapping Department, Agricultural Genetic Engineering Research Institute (AGERI), Agricultural Research Center (ARC), Giza 12619, Egypt; matia@ageri.sci.eg (M.A.M.A.)
[5] Medical Biochemistry Department; National Research Centre, 33 El Bohouth St., Dokki, Giza 12622, Egypt; karammahdy64@gmail.com
[6] Department of Biology, College of Science, University of Hafr Al Batin, Hafar Al Batin, Saudi Arabia; Dr.shifaa@uhb.edu.sa
[7] Department of Pharmaceutical Sciences, College of Pharmacy & Thumbay Research Institute for Precision Medicine, Gulf Medical University, P.O. 4184, Ajman, United Arab Emirates; dr.alabd@gmu.ac.ae
[8] Pharmacology Department, Medical Division, National Research Centre, Cairo, Egypt; ahmedmalabd@pharma.asu.edu.eg
[9] Department of Biology, College of Science, King Khalid University, Abha 9004, Saudi Arabia; hamdony@yahoo.com
[10] Department of Botany & Microbiology, Faculty of Science, South Valley University, Qena, Egypt
[11] Pathology Department; National Research Centre, 33 El Bohouth St., Dokki, Giza 12622, Egypt; ar.hussein@nrc.sci.eg

- Correspondence: ahmedmalabd@pharma.asu.edu.eg; Tel.: +971-56-464 2929 (A.M.A-A.)

Supporting data **Page**

S1:^{1}H NMR (CD_3OD, 600 MHz) of ST1 ……………………….. 2

S2:^{13}C NMR (CD_3OD, 150 MHz) of ST1 ……………………… 3

S3:^{1}H ^{1}H COSY of ST1…………………………………………… 4

S4:HMBC of ST1……………………………………………………. 5

S1: ^{1}H NMR (CD_3OD, 600 MHz) of ST1

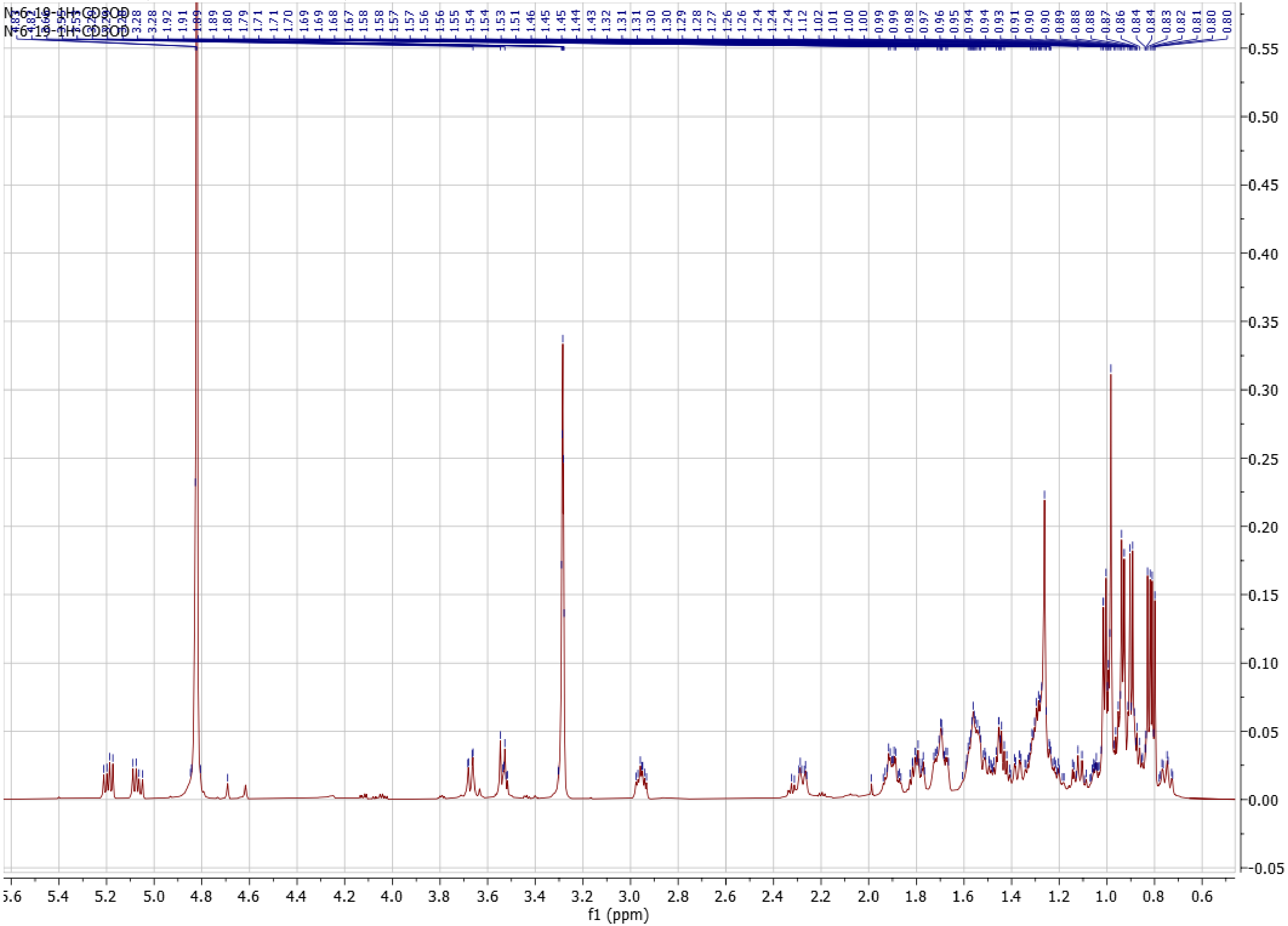

S2: ^{13}C NMR (CD_3OD, 150 MHz) of ST1

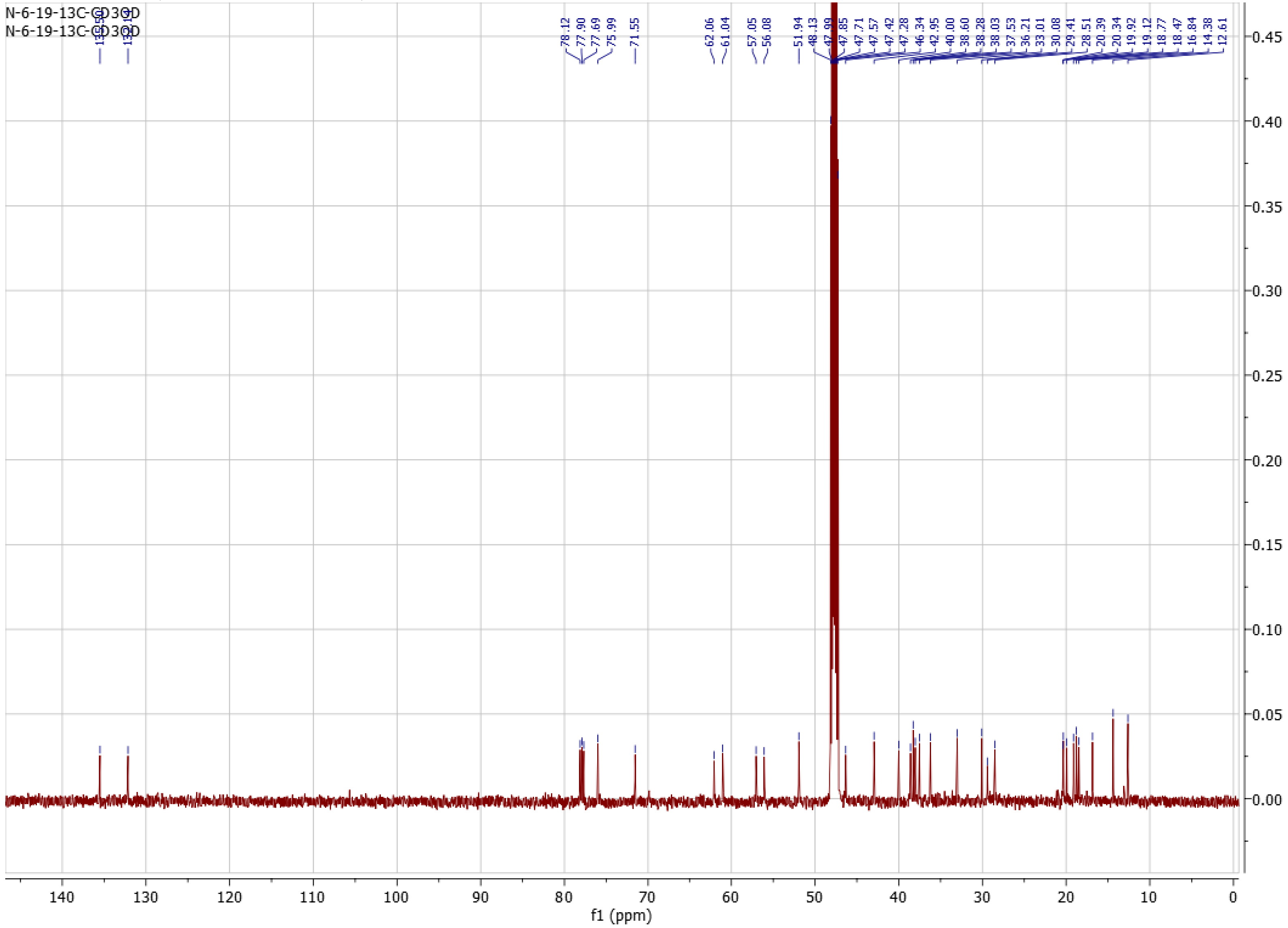

S3: ^{1}H ^{1}H COSY of ST1

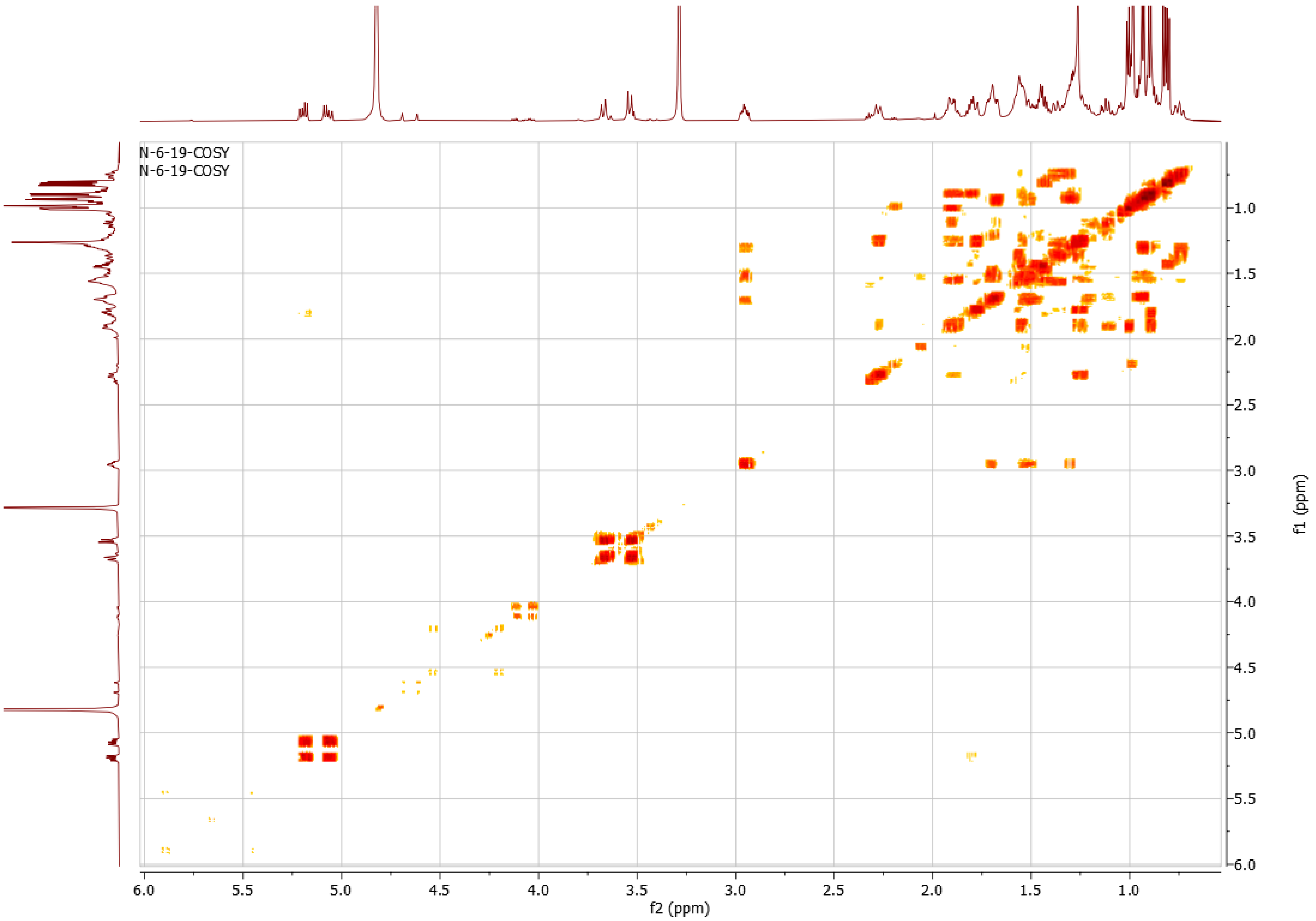

S4:HMBC of ST1

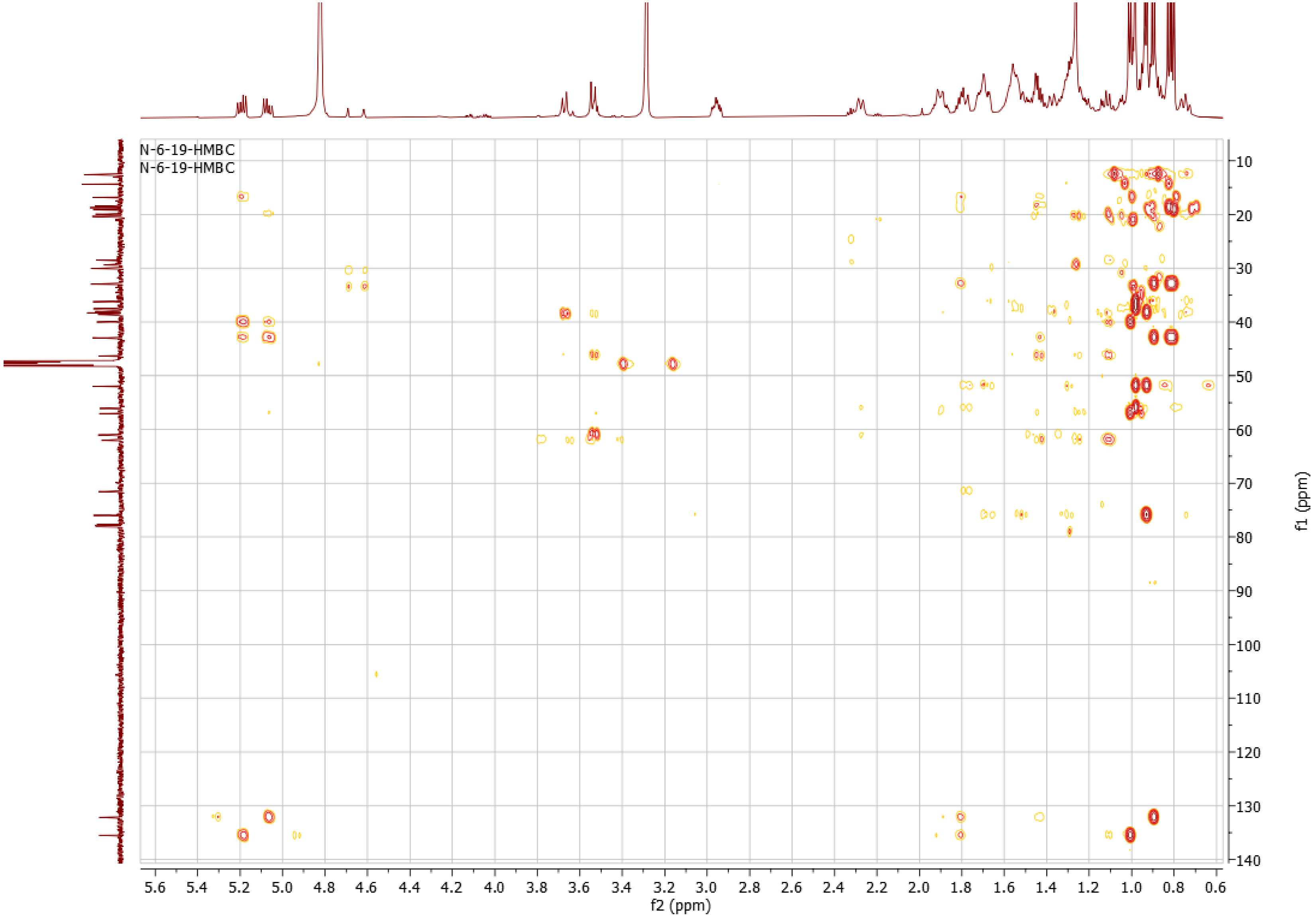

Supplement: Supplementary file 1 [file biomolecules-11-01247-s001.zip › biomolecules-1297250-supplementary.pdf]
